# Tracking the Deer Tick: Emerging Lyme Disease Threat in Canada

**DOI:** 10.1289/ehp.118-a305a

**Published:** 2010-07

**Authors:** Tanya Tillett

**Affiliations:** **Tanya Tillett**, MA, of Durham, NC, is a staff writer/editor for *EHP*. She has been on the *EHP* staff since 2000 and has represented the journal at national and international conferences

Lyme disease was first diagnosed during an outbreak in the Northeast and upper Midwest of the United States in the late 1970s. Since that time, the disease has become well established in the northeastern United States. A new study now indicates Lyme disease is continuing to spread north into Canada and, because of a convergence of environmental factors, is poised to emerge as a potential public health threat in southern Quebec **[*EHP*** **118(7):909–914; Ogden et al.]**.

Caused by the bacterium *Borrelia burgdorferi*, Lyme disease is spread through the bite of the deer tick (*Ixodes scapularis*). Symptoms can include skin rash, joint pain, fatigue, and more serious neurologic disorders if the disease is left untreated. Until recently, cooler climate patterns in Canada did not favor the infiltration of *I. scapularis* and consequent spread of Lyme disease, but a warmer climate in southern Quebec may be easing the way for “adventitious” ticks—nonnative ticks introduced most likely by migratory birds—to become established.

In the current study, researchers analyzed data for *I. scapularis* presence and *B. burgdorferi* infection based on passive surveillance (that is, ticks collected voluntarily by medical and veterinary clinics in Quebec were submitted to the provincial public health laboratory) and active surveillance (the research team’s own field analysis of 71 woodland sites in three regions of southern Quebec) to identify areas where Lyme disease is emerging.

*I. scapularis* have been collected through passive surveillance in Quebec since 1990, but the investigators observed that between 2004 and 2008 the number of ticks collected increased exponentially to more than 1,700 per year. Given that no marked increase occurred in the number of participating clinics during this time, the increase suggests that in addition to the presence of adventitious ticks, breeding populations of *I. scapularis* have now become established in the region. The authors observed a *B. burgdorferi* infection rate of 13.2% in ticks collected through passive surveillance but a lower prevalence of infection in ticks collected at the active surveillance sites (7.7% overall), implying the *I. scapularis* populations that are becoming established are initially free of *B. burgdorferi*. Ticks that did carry *B. burgdorferi* carried strains that were mostly identical to those seen in the northeastern United States.

The authors postulate that warming climate conditions, a growing tick population, and infected ticks hitchhiking from the United States have set up a favorable scenario for increasing the threat of Lyme disease in southern Quebec. They write that increased surveillance in Quebec and the rest of southeastern Canada would help track the progression of risk areas and protect public health.

## Figures and Tables

**Figure:**
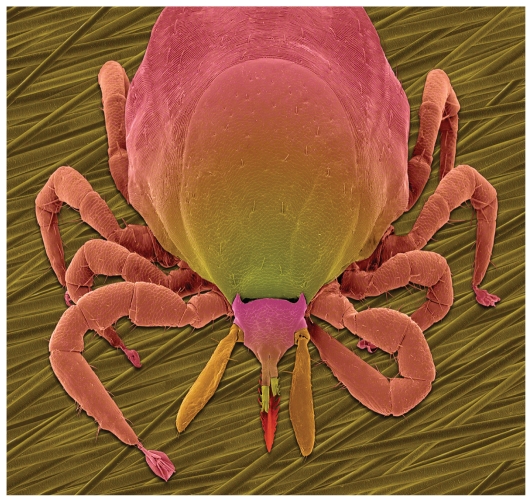
Deer tick (*Ixodes scapularis*)

